# Inactivation of Human Coronavirus by FATHHOME’s Dry Sanitizer Device: Rapid and Eco-Friendly Ozone-Based Disinfection of SARS-CoV-2

**DOI:** 10.3390/pathogens10030339

**Published:** 2021-03-14

**Authors:** Timsy Uppal, Amir Khazaieli, Antoine M. Snijders, Subhash C. Verma

**Affiliations:** 1 Department of Microbiology and Immunology, Reno School of Medicine, University of Nevada, 1664 N Virginia Street, Reno, NV 89557, USA; tuppal@med.unr.edu; 2 FATHHOME, INC., 8000 Edgewater Drive Suite #200, Oakland, CA 94621, USA; amirk@fathhome.net; 3 Lawrence Berkeley National Laboratory, Biological Systems and Engineering Division, 1 Cyclotron Road, Berkeley, CA 94720, USA; amsnijders@lbl.gov

**Keywords:** FATHHOME, SARS-CoV-2, COVID-19, virus decontamination, PPE

## Abstract

The pandemic of SARS-CoV-2/COVID-19 was reported in December 2019 in Wuhan, China. Pertaining to its high transmissibility and wide host adaptability, this unique human coronavirus spread across the planet inflicting 115 million people and causing 2.5 million deaths (as of March 3rd, 2021). Limited or negligible pre-existing immunity to multiple SARS-CoV-2 variants has resulted in severe morbidity and mortality worldwide, as well as a record-breaking surge in the use of medical-surgical supplies and personal protective equipment. In response to the global need for effective sterilization techniques, this study evaluated the virucidal efficacy of FATHHOME’s self-contained, ozone-based dry-sanitizing device, by dose and time response assessment. We tested inactivation of human coronavirus, HCoV-OC43, a close genetic model of SARS-CoV-2, on porous (N95 filtering facepiece respirator/FFR) and nonporous (glass) surfaces. We started our assays with 20 ppm-10 min ozone exposure, and effectively reduced 99.8% and 99.9% of virus from glass and N95 FFR surfaces, respectively. Importantly, the virus was completely inactivated, below the detection limit (over 6-log_10_ reduction) with 25 ppm-15 min ozone exposure on both tested surfaces. As expected, a higher ozone exposure (50 ppm-10 min) resulted in faster inactivation of HCoV-OC43 with 100% inactivation from both the surfaces, with no residual ozone present after completion of the 5-min post exposure recapture cycle and no measurable increase in ambient ozone levels. These results confirmed that FATHHOME’s device is suitable for rapid decontamination of SARS-CoV-2- from worn items, frequently touched items, and PPE including N95 FFRs, face shields, and other personal items.

## 1. Introduction

The recently identified SARS-CoV-2, a *betacoronavirus* of zoonotic origin [1], has been implicated as the etiologic agent of the COVID-19 pandemic [2]. COVID-19 is a severe and potentially fatal infectious disease that can be spread by asymptomatic, pre-symptomatic, and symptomatic carriers [3,4]. As of 3rd March 2021, over 115 million people worldwide [5] have been infected with SARS-CoV-2, threatening human health and public safety. Even though a large portion of COVID-19 patients remain asymptomatic or mildly symptomatic, the infection causes serious complications in susceptible individuals [6]. COVID-19 disease is characterized by complications including endothelial barrier disruption, dysfunctional alveolar-capillary oxygen transmission, and impaired oxygen diffusion capacity [7]. In critical symptomatic cases, life threatening acute respiratory distress syndrome (ARDS) associated with hypoxemic respiratory failure and non-cardiogenic pulmonary edema is the main cause of death [8,9,10]. SARS-CoV-2 mediated endothelial tissue injury is believed to cause alveolar permeability disruption and pulmonary vascular thrombosis in COVID-19 patients with ARDS [11].

Infections associated with the 2019 SARS-CoV-2 outbreak spread by contact, respiratory droplet, and airborne transmission [12]. Surfaces of animate and inanimate objects have been reported to contribute in the spread of SARS-CoV-2 infections [13,14]. The presence, stability, and infectivity potential of SARS-CoV-2 has been reported for different types of clinical samples (urine, sputum, blood, feces, and bronchoalveolar fluid) [15,16], on different surfaces (floor, door handle, bed rail, bedside table, microwave oven/closet/faucet handle, mobile phone, eyeglasses) [17,18], and on a variety of surface materials (metal, rubber, ceramic, surgical glove, wood, cloth, plastic, stainless steel, surgical mask, and tissue paper) [13]. SARS-CoV-2 is viable on inanimate surfaces, one of the most prone sites for the virus transmission, for prolonged times estimated to be between 2 h and 9 days, with virus persistence depending on the temperature, pH, relative humidity, and nature of the surface [19]. Although vaccinations and anti-viral therapies are given the top priority to reduce the spread of COVID-19 disease, according to WHO guidelines, effective infection prevention and control (IPC) is a practical, evidence-based approach to restrict disease spread [12].

Current interventions for COVID-19 prevention include an effective surface disinfectant, appropriate hand hygiene, and personal protective equipment (PPE), primarily suitable face mask/respirator, gloves, face shield, and gowns. According to United States Environmental Protection Agency (USEPA), effective surface disinfectants with significant virucidal activity at low contact time, mostly contain hydrogen peroxide, peroxyacetic acid, 70% ethanol, phenol, quaternary ammonium salt derivatives, aldehyde, hypochlorous acid, sodium hypochlorite, sodium bicarbonate, octanoic acid, citric acid, conjugates with silver ions, as the key active ingredients [12,20]. Kampf and Colleagues recently analyzed 22 research reports that studied inactivation of SARS and MERS (human coronaviruses/HCoVs of epidemic potential) with biocidal agents, and revealed that surface disinfection using 62–71% ethanol, 0.5% hydrogen peroxide, or 0.1% sodium hypochlorite inactivated the HCoVs within one minute [14]. Several methods, for instance, high temperature (including autoclaving at 121 °C) [21,22], heat [23,24], sunlight [25], 70% ethanol [26,27], microwave irradiation [28], detergents [23], Trizol reagent [23], ozone [29], vaporized hydrogen peroxide [30], and gamma/UV radiations [31,32,33] have been implemented for rapid inactivation of SARS-CoV-2-contaminated surfaces. In 2017, the WHO developed and recommended two alcohol-based hand rub formulations, WHO-I (80% ethanol-1.45% glycerol-0.125% hydrogen peroxide) and II (75% isopropyl alcohol-1.45% glycerol-0.125% hydrogen peroxide) against Ebola virus, human Influenza A virus, and Modified vaccinia Ankara strain [34]. In a study by Kratzel et al., both of these hand rub formulations demonstrated potent virucidal activity against SARS-CoV-2, providing another effective tool for individual preventive care [26]. Consistent and robust evidence from many studies emphasizes that the use of respirators decreased the risk of infection in SARS-CoV-2 [35,36]. Several federal agencies, including the Center for Disease Control and Prevention (CDC), National Institute for Occupational Safety and Health (NIOSH), Food and Drug Administration (FDA), and Occupational Safety and Health Administration (OSHA), recommended the use of surgical masks and N95 filtering facepiece respirators (FFRs), along with gowns, eye protection, and gloves to prevent the spread of respiratory SARS-CoV-2 infection in the health care setting [37].

In response to the global COVID-19 pandemic and dwindling supplies of PPE [38], the WHO recommended a 40% increase in the production of PPE, especially surgical masks and N95 FFRs [12]. Increased demand for surgical masks, N95 FFRs, and other PPE has resulted in a need for repeated usage of single-use disposable PPE and routine decontamination, as a crisis capacity strategy, with focus towards disinfection technologies [38]. Although FFRs are essential to protect from airborne viruses, the use of a contaminated FFR could potentially serve to self-inoculate or spread the virus to patients or other healthcare workers. Existing decontamination strategies of PPE include ultraviolet germicidal irradiation (UVGI) [33,39,40], heat sterilization [41,42], chemical disinfectants [43], microwave radiation [40,41], and vapor phase hydrogen peroxide (VHP) [44,45]. While these methods can effectively reduce pathogen load, material damage such as that caused by heat sterilization, peroxide vapor treatment, microwave irradiation, and chemical disinfectants may result in poor fit, reduced filtration efficiency, and breathability, which will in turn increase the wearer’s potential exposure to pathogens [33,46,47]. This leads to a demand for standardized, sustainable decontamination systems.

In this context, we sought to evaluate the efficacy of FATHHOME’s ozone-based dry disinfection device towards inactivation of HCoV-contaminated PPE. Given the high similarity of HCoV-OC43 to SARS-CoV-2 (BSL-3+ pathogen), lower infectious risk, and presence of lower-containment laboratory, we used HCoV-OC43 as a SARS-CoV-2 surrogate, to determine the efficacy of ozone gas in virus inactivation [48]. HCoV-OC43 (Biosafety level-2+/BSL-2+ pathogen), is a *betacoronavirus* that causes respiratory illness among infected individuals [49]. We optimized the conditions for disinfecting HCoV-contaminated PPE by varying the ozone exposure time and ozone concentration. Residual intact viral particles, post treatment was detected, using qRT-PCR to quantify the viral genomes. Furthermore, qRT-PCR and immunofluorescence were used to detect residual infectious virus after infection of ACE2 expressing A549 cells.

## 2. Materials and Methods

### 2.1. Cells

The human lung adenocarcinoma NCI-H441 cells were purchased from ATCC and maintained in RPMI medium supplemented with 10% fetal bovine serum (FBS, Atlanta Biologicals, Flowery Branch, GA, USA), 2 mM L-glutamine, 25 U/mL penicillin, and 25 μg/mL streptomycin. The A549-hACE2 (HA-Flag) cells were obtained from BEI Resources (NIAID, NIH) and maintained in Dulbecco’s modified Eagle medium (DMEM) supplemented with 10% FBS (Atlanta Biologicals), 2 mM L-glutamine, 25 U/mL penicillin, and 25 μg/mL streptomycin and 1 μg/mL puromycin. The cells were grown at 37 °C and 5% CO_2_ in a humidified chamber.

### 2.2. Human Coronavirus

HCoV-OC43 strain, is a human coronavirus and belongs to the family *Coronaviridae*, genus *betacoronavirus*. HCoV-OC43 was obtained from BEI Resources and propagated in NCI-H441 cells by infecting the cell monolayer with the virus for 2 h at 34 °C. Unattached virus was removed by washing followed by addition of fresh medium. After 4 days, supernatant containing virus was harvested, cell debris were removed by centrifugation, and the virus was aliquoted and stored at −80 °C until further use. Viral copies in the harvested supernatant were quantified by Reverse Transcriptase qPCR (qRT-PCR) using a standard curve (described later in Section 2.3) as well as infectivity assay and the detection of virally infected cells through immunofluorescence assay (IFA). All the assays were performed under BSL-2+ containment.

### 2.3. RNA Extraction and qRT-PCR

For the detection of viral genomic RNA through qRT-PCR, control or ozone-treated virus from the supernatant (for intact viral genomic RNA) or from infected A549-hACE2 (HA-FLAG) cells (for infectious viral genomic RNA) were subjected for total RNA extraction using Trizol reagent (Invitrogen, Carlsbad, CA, USA), according to the manufacturer’s recommendation. An aliquot of extracted total RNA (1 μg) was used for synthesizing the cDNA using high-capacity RNA to cDNA kit (Invitrogen). A fraction of synthesized cDNA (5 μL) was used for the relative quantification of viral genomic copies using OC43-SF 5′-GGCTTATGTGGCCCCTTACT-3′ and OC43-SR 5′-GGCAAATCTGCCCAAGAATA-3′ primer set in a qRT-PCR assay (Thermo Fisher Scientific, Waltham, MA, USA). Tenfold serial dilutions of HCoV-OC43 genomic RNA (BEI Resources, cat. # NR-52727 with 2.0 × 10^8^ genome equivalents/mL) were used for generating a standard curve to quantify the HCoV-OC43 viral copies in the virus preparations.

### 2.4. Virus Infectivity through Immunofluorescence Assay

Control or ozone-treated viruses were added onto the human lung carcinoma, A549-hACE2 (HA-FLAG) cells for 2 h (34 °C, 5% CO_2_). Following infection, the cells were incubated for 48 h at 34 °C in a humidified chamber supplemented with 5% CO_2_. Infected cells were detected by using IFA of the nucleocapsid protein of HCoV-OC43 (Millipore Sigma, Burlington, MA, USA). Infected cells were fixed using 3.2% formaldehyde and permeabilized with 0.1% Triton X-100 for 10 min, washed (2x) and blocked (0.4% FSG-0.05% Triton X-100) for 45 min at room temperature. Cell monolayers were washed (2x) and incubated with monoclonal mouse anti-HCoV-OC43 nucleocapsid antibody (1:1000; Millipore Sigma) overnight at 4 °C, followed by incubation with chicken anti-mouse Alexa Fluor 594 (1:1000; Invitrogen), secondary antibody for 1h at RT in the dark. Finally, the nuclei were stained with DAPI (1:7000; Thermo Fisher Scientific). Coverslips were mounted on the glass slides using prolong diamond antifade (Thermo Fisher Scientific) and the slides were examined using Carl Zeiss LSM 780 microscope.

### 2.5. FATHHOME Device

FATHHOME’s ozone-based dry sanitizer is equipped with a computer-controlled ozone generator and a catalytic manganese dioxide-copper oxide (MnO_2_-CuO_2_) converter which, along with the negative pressure chamber comprises a self-contained gas-based sanitizing system. The system maintains virucidal levels of sanitizing gas while cycling internal pressures between −30 kPa and −15 kPa, which permeates ozone throughout the chamber while ensuring none of it escapes during the sanitization cycle. Once the vacuum seal is generated, ozone (O_3_) is produced by the device at point-of-use using electricity and atmospheric air, through cleavage of O_2_ into elemental oxygen (which combines with molecular oxygen (O_2_) to create transient O_3_) via a corona discharge device. The system then back-fills the evacuated chamber with air while an onboard microcontroller attempts to maintain target ozone concentration levels (20 ppm, 25 ppm, 50 ppm). At all times, the contents of the chamber are being held under negative pressure with the only exit for all gases in the system being pumped through the MnO_2_-CuO_2_-O_3_ scrubber to ensure environmentally safe device exhaust within OSHA and FDA guidelines. The FATHHOME device can be operated through its onboard microcontroller, which is accessed either via a switch located on the device or via a cloud-based web-panel. For the duration of this study, the FATHHOME device was kept inside BSL-2+ containment and controlled remotely outside the test room for each disinfection cycle.

### 2.6. Experiment Set up

**Figure 1:**
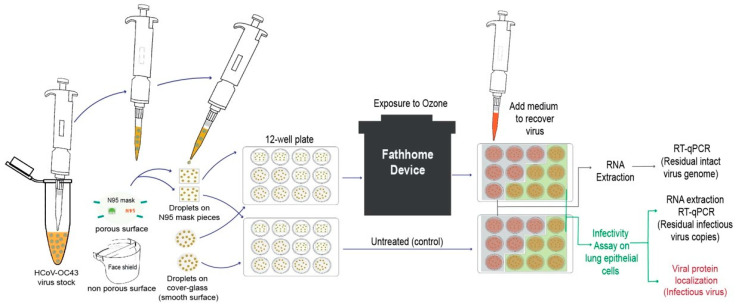
Schematic representation of the experimental set up utilized for the evaluation of ozone-based inactivation of HCoV-OC43 using FATHHOME device. An aliquot (100 µL) with known amounts of HCoV-OC43 virions was placed on the chosen surface (glass coverslips and N95 FFR) and exposed to ozone for indicated exposure dose and time. The mock- and ozone-treated viruses were then collected to determine the viral RNA stability (via quantitative RT-PCR), and viral infectivity, which involves detection of infectious intracellular RNA copies (via qRT-PCR) and localization of viral protein (through IFA) in A549-hACE-2 (HA-FLAG) cells.

In order to test the efficacy of ozone in accelerating the inactivation of HCoV-OC43 using FATHHOME’s dry sanitizing device, 100 µL of the virus stock (equivalent to 2.1 × 10^6^ calculated viral particles) were applied as 10 × 10 µL liquid droplets on top of glass coverslips (Thermo Fisher Scientific, 18 mm diameter), and N95 FFRs (Thermo Fisher, 1 cm^2^ square pieces), each placed in the center of a well of 12-well culture plate (Figure 1), kept inside the BSL-2+ containment.

The applied virus was present in the culture medium containing fetal bovine serum, providing proteinaceous content resembling the composition of nasal secretions or respiratory droplets. The virus-containing 12-well plate was placed inside the FATHHOME device (at the center) and exposed to varying doses of ozone gas (20, 25 and 50 ppm) for specified times (10, 15, and 20 min). The sanitizing chamber’s ozone-gas concentration level was monitored during each disinfection cycle using a NIST Calibrated ozone sensor (FD-600-O_3_ Ozone Analyzer, Forensics Detectors, Rolling Hills Estates, CA, USA) connected directly to a 3/32” sensor port on the FATHHOME device’s vacuum chamber. The FD-600-O_3_ features a built-in extraction pump that allows it to detect 0–100 ppm ozone with 0.1 ppm resolution under both atmospheric and vacuum pressure conditions. These data were logged to csv over a Serial-USB interface at a sampling rate of 1 measurement per second. Any residual ozone gas leakage during the ozone disinfection cycle was measured (on-screen) using a second ozone gas sensor (Porta Sens II, Thermo Fisher Scientific) placed in the BSL-2 + containment next to the FATHHOME device. All the experiments were conducted at 25 °C and 45% relative humidity (standard indoor humidity), as measured with AcuRite Indoor Thermometer and Hygrometer with Humidity Gauge (AcuRite, Inc., Lake Geneva, WI, USA). Virus-laden glass coverslips and N95 FFRs without ozone treatment served as a control for calculating viral inactivation efficiencies. After each disinfection cycle, the mock-treated and ozone-treated viruses were recovered by adding 500 μL of culture medium on the surfaces and allowing it to resuspend, for 15 min at 34 °C. For viruses applied to porous N95 FFRs, where the virus could be absorbed into multiple layers, any remaining virus was recovered by incubating the treated material with 500 μL of culture medium for 15 min at 34 °C, followed by centrifugation at 12 K rpm for 5 min, and removal of N95 FFR material. The recovered viruses were collected in a 1.5 mL eppendorf tube either for further extraction of total viral RNA with Trizol reagent for viral genome quantitation using qRT-PCR or applied onto a permissive A549-hACE2 HA-FLAG cell monolayer for the detection of residual infectious virus through integrated cell culture PCR and localization of infected cells through immunofluorescence assay (Figure 1). The A549-hACE2 (HA-FLAG) cells are a human lung carcinoma (A549) cell line over-expressing human angiotensin converting enzyme-2 (hACE-2) under control of the XYZ promoter. Each assay was done in duplicates and each experiment was conducted three independent times.

### 2.7. Statistical Analysis

Data presented are an average of three independent experiments and the error bars represent the standard deviation across independent experiments. Statistical analyses were performed using Prism 8.0 software (Graphpad Inc., San Diego, CA, USA) and the *p*-values were calculated using 2-way ANOVA and the *p*-value cut offs for statistical significance were *, *p* < 0.1; and **, *p* < 0.01.

## 3. Results

### 3.1. Assay Development to Quantify Infectious HCoV-OC43

**Figure 2:**
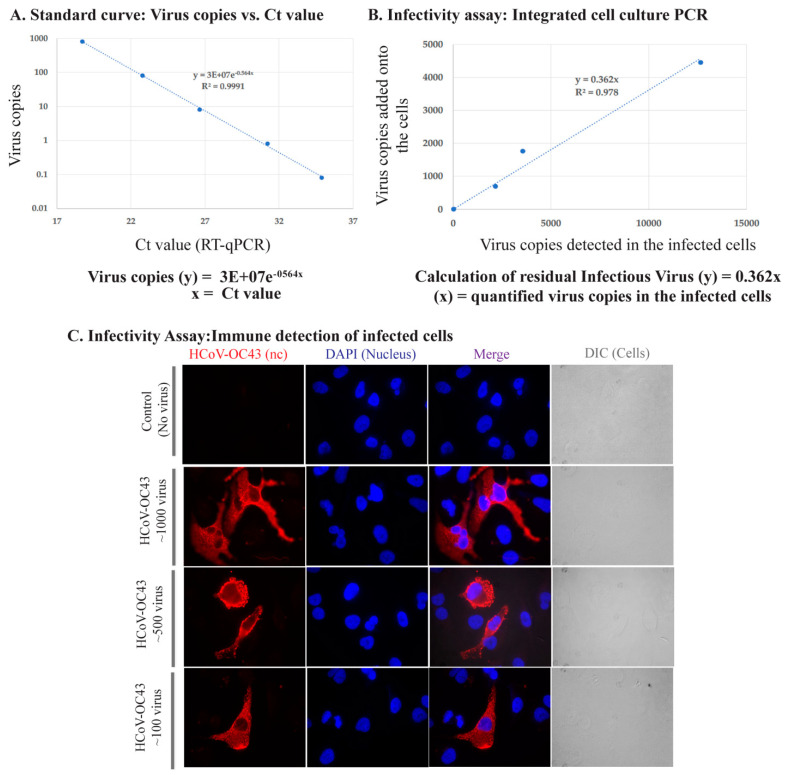
Assay establishment: Integrated cell culture PCR and Immune detection of HCoV-OC43 infected cells. (**A**). Standard curve of varying HCoV-OC43 genome copies with Ct value. (**B**). Correlation of HCoV-OC43 viral copies added onto A549-hACE2 cells and detection of intracellular viral genome copies following infection and replication. (**C**). Immune localization of HCoV-OC43 nucleocapsid protein in A549-hACE2 cells infected with varying amounts of virus. Nucleocapsid was detected with anti-HCoV-OC43 antibody followed by staining with chicken anti-mouse Alexa Fluor 594 (red). Nuclei were stained with DAPI (blue). DIC images are to show cell morphology.

In order to quantify infectious virus, we utilized an integrated cell culture PCR assay and the detection of infected cells by quantifying viral genomic RNA and by localizing HCoV-OC43 nucleocapsid protein through immunofluorescence of infected cells. Varying amounts of HCoV-OC43 virus, calculated using a standard curve (Figure 2A) from our stock, were added onto a permissive cell line, A549-hACE2 (infectivity assay) followed by the detection of viral genome copies after a 48 h incubation. We detected a strong correlation between the amount of viral genome copies detected and the amount of virus copies added onto the cells (R^2^ = 0.978) (Figure 2B). We also confirmed the infectivity of our virus stock and the detection of viruses in the infected cells by adding different quantities of viral particles on A549-hACE2 and localizing HCoV-OC43′s nucleocapsid using immunofluorescence antibody staining. Lack of any signals in control (uninfected cells) and specific localization of nucleocapsid signals confirmed the specificity of our assay (Figure 2C). Further, cells infected with lower amounts of HCoV-OC43 virus showed reduced number cells with nucleocapsid detection, as expected (Figure 2B).

### 3.2. Effect of Ozone Exposure on HCoV-OC43 Virus Stability on Different Surfaces

**Figure 3:**
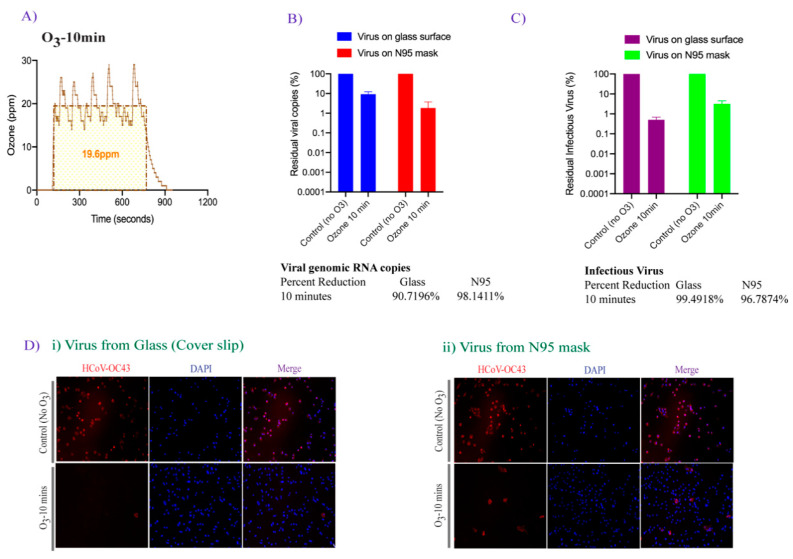
The effect of 20 ppm-10 min ozone exposure on HCoV-OC43 viral genomic RNA stability and infectivity. (**A**). The plot depicting the average ozone dose (20 ppm-10 min) generated via FATHHOME device to which the virus was exposed. The ozone dose data were recorded using the connected FD-600-O_3_ ozone detector. (**B**). HCoV-OC43 virus (10 × 10 µL droplets) was applied on coverslips and N95 FFRs and exposed to 20 ppm ozone gas for 10 min. Total RNA was extracted and used for the detection of viral copies by qRT-PCR. (**C**). HCoV-OC43 virus (100 µL) was exposed to 20 ppm-10 min ozone treatment, collected, and used for the infection of A549-hACE-2 cells. Total RNA was extracted 48 h post-infection for the detection of intracellular viral genomic copies in a qRT-PCR assay. Viral copies were calculated based on a standard curve generated using the known amounts of virus (BEI Resources), and quantified with respect to untreated controls, set to 100%. (**D**). Untreated and ozone-treated HCoV-OC43 virus infected A549-hACE-2 cells were fixed and immune stained with HCoV-OC43 antibody (for nucleocapsid protein) for immunofluorescence assay. Virus internalization showed virus preferably localized on the membrane (red signals) in HCoV-OC43 infected cells. Nuclei were stained with TO PRO-3 (blue signals). The uninfected and infected cells were identified and quantified using automated Image J macro method.

The FATHHOME dry sanitization device is based on antimicrobial ozone technology, and has been previously shown to inactivate more than 99% of *E. coli* bacterium on contaminated fabric [50]. In an attempt to test the disinfection efficiency of FATHHOME on HCoVs, HCoV-OC43 was used as a surrogate of the highly contagious SARS-CoV-2 for practical reasons. Given the high genomic sequence similarities between *betacoronavirus* family members, human cell entry and infection mechanisms as well as inactivation conditions are likely to be similar [51]. In this study, we tested the virus decontamination on two different surfaces, i.e., a nonporous smooth surface (glass coverslips) and a porous, soft surface (N95 FFRs). HCoV-OC43 virus suspensions (10 × 10 µL liquid droplets) were applied onto each surface and placed inside a 12-well plate. The plate containing HCoV-OC43 viruses was exposed, after removing the lid, to an ozone concentration of approximately 20 ppm and an ambient pressure of 68 kPa to 85 kPa produced by the FATHHOME device, situated inside a BSL-2+ containment for 10 min (Figure 3A). Glass coverslips and N95 FFRs containing the same amount of virus without ozone treatment were used as a control in our assay. Untreated and ozone-treated viruses were collected for the evaluation of virus inactivation by direct genomic RNA quantitation (qRT-PCR) and infectivity assay using permissive A549-hACE2 cells. Presence of the viral genomic RNA was determined using qRT-PCR as used earlier for detection of SARS-CoV-2 in environmental surface and air samples [17]. Detection of genomic viral RNA through qRT-PCR is considered to be a highly sensitive method for the detection of viral genomic RNA, however a positive result does not necessarily mean that viral genomic RNA is intact or that the RNA is inside an infectious virus. However, absence of a virus specific PCR signal confirms absolute removal of virus and viral genome. Therefore, viral genomic RNA quantitation along with infectivity assays was devised for estimating viral inactivation efficacies. HCoV-OC43 viral copies in the ozone treated and untreated samples were calculated based on the standard curve generated using serial dilutions of known HCoV-OC43 genomic RNA (Figure 2). The intact residual virus copies in ozone-treated samples were calculated and compared to the untreated controls, set to 100%. As expected, the copies of viral genomic RNA declined (i.e., one log_10_ decrease) after 10 min, 20 ppm ozone-exposure on both the tested surfaces (Figure 3B). Interestingly, we saw a slightly higher reduction (98.14%) in the viral genomic RNA on N95 FFRs than glass coverslips (90.71%) in this assay (Figure 3B).

In order to determine residual infectious virus, untreated control and ozone exposed HCoV-OC43 viruses were added onto the A549-hACE2 cells for 2 h at 34 °C to facilitate attachment and entry of the residual live virus. The over-expression of ACE-2, the entry receptor for HCoVs including, HCoV-NL63, SARS-CoV, and SARS-CoV-2, facilitates the entry of HCoV-OC43 into the cells [52]. Quantitation of intracellular HCoV-OC43 genome copies through spike glycoprotein gene specific qRT-PCR led to the calculation of infectious virus added onto the target cells as described in Figure 2B. Calculated residual live virus copies based on the intracellular viral genomic copies, showed that ozone effectively reduced the amounts of infectious virus when compared with the control, non-ozone treated samples. Noticeably, there was a higher, 99.49% reduction (Figure 3C), in infectious virus on smooth surface than from soft surface (96.78% reduction). These observed differences could be attributed to surface composition variability, which affects virus adsorption and infectivity. Additionally, presence of live virus was detected by localizing the viral nucleocapsid protein through immunofluorescence staining for HCoV-OC43 viral protein (Figure 3D). As shown in Figure 3D, the addition of ozone-exposed HCoV-OC43 virus resulted in a reduction of the virus-positive cells, compared to the untreated controls where 95% of cells were positive for HCoV-OC43 protein. In contrast, for ozone-exposed virus, we only observed 5 and 14 cells stained positively for HCoV-OC43 protein (out of 500 cells) after virus recovery from the glass and N95 FFRs surfaces, respectively. Overall, these results indicate that ozone is effective in inactivating human coronavirus.

### 3.3. Effect of Increased Contact Time of Ozone Exposure on HCoV-OC43 Virus Inactivation

**Figure 4:**
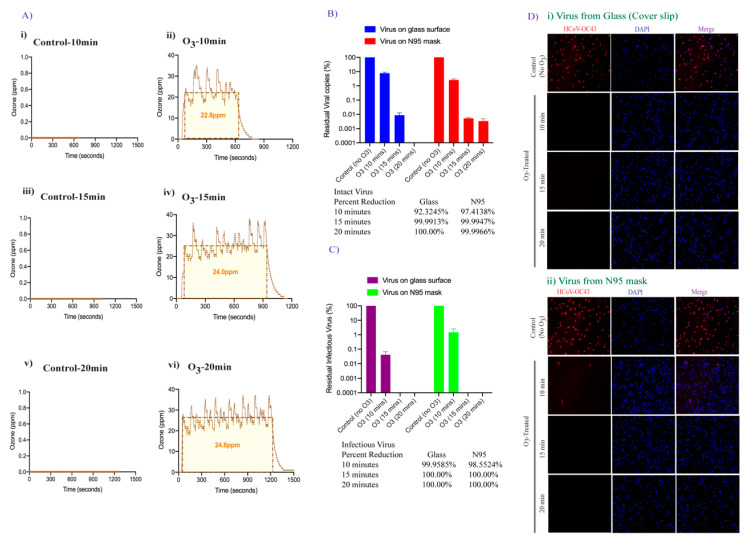
FATHHOME’s ozone dose-response assessment of HCoV-OC43 inactivation. (**A**). The ozone (ppm) vs. time (sec) plot showing the average ozone gas to which the virus was exposed during ~25 ppm-10, 15, and 20 min ozone treatment, as generated via FATHHOME device. (**B**). HCoV-OC43 virus aliquot (100 µL) was placed on glass coverslips and N95 FFRs and exposed to 25 ppm of ozone for 10, 15-, and 20-min. Following ozone treatment, HCoV-OC43 was collected for viral RNA extraction. Intact residual viral copies were calculated based on the standard curve. (**C**). HCoV-OC43 virus was collected post ozone treatment, for virus infectivity and replication, and subjected to the infection of A549-hACE-2 cells. Intracellular infectious RNA copies were determined through qRT-PCR and viral copies were calculated based on the standard curve and quantified with respect to untreated controls, set to 100%. (**D**). HCoV-OC43 infection and replication in untreated and ozone-treated HCoV-OC43 virus infected cells was detected by immune localization of HCoV-OC43 nucleocapsid protein through IFA (red signals). Nuclei were stained with TO PRO-3 (blue signals). The quantification of cells was done using automated Image J macro analysis.

We next examined the possibility of improving viral inactivation and viral genome degradation by exposing the surfaces containing virus containing droplets to increasing levels of ozone and exposure time. The surfaces were exposed to 25 ppm ozone and an ambient pressure of 68 kPa to 85 kPa for 10, 15, and 20 min (Figure 4A). The recovered virus was either evaluated for viral genome RNA stability (using qRT-PCR) or added onto the cells for residual infectious virus quantification using qRT-PCR and immunofluorescence assay as described above. Surprisingly, an increase in ozone exposure to 25 ppm for 10 min did not significantly change the number of intact viral genomic copies on both smooth and porous surfaces (Figure 3 and Figure 4 panel B). In contrast, increasing the ozone contact time (from 10 to 15 min) dramatically enhanced the degradation of HCoV-OC43 virus (4-fold, 99.99% reduction) and a further increase in the contact time (from 15 to 20 min) led to complete degradation of viral genomic RNA on smooth surfaces (Figure 4B). For N95 FFRs, increasing the contact time of 25 ppm ozone exposure from 10 to 15 min, also resulted in a 99.99% HCoV-OC43 reduction, and increasing exposure time to 20 min did not further decrease viral genomic RNA (Figure 4B). Since the qRT-PCR detects any traces of viral genome even from already inactivated virus particles, we further determined the copies of infectious virus using the infectivity assay. Recovered virus after ozone treatment were added onto A549-hACE2 cells for residual infectious virus to infect the cells and replicate for 48 h. Estimation of residual infectious viral copies for 25 ppm-10 min ozone treatment, through qRT-PCR confirmed lower copies of the infectious virus (three log_10_ reduction) were recovered from smooth surface as compared to virus recovered from soft surface (one log_10_ reduction, Figure 4C). Further, the HCoV-OC43 virus had been completely degraded (below the detection limit) on both the surfaces after 15- and 20-min exposure of 25 ppm ozone. These results confirmed that surface disinfection of coronavirus can be achieved quickly and safely, and that ozone dose and contact time can further enhance the sterilization activity. The results from the infectivity assay, detecting HCoV-OC43 infected cells, with ozone-treated and untreated virus correlated well with the qRT-PCR results (Figure 4D). Absence of any HCoV-OC43 protein stained A549-hACE2-cells, as compared to untreated control virus, further confirmed complete HCoV-OC43 inactivation 10, 15, and 20 min after 25 ppm-ozone treatment on smooth surface. Although we detected 1.6% HCoV-OC43 protein positive cells (8 positive cells out of 500 cells examined) in virus recovered from N95 mask exposed 10 min after 25 ppm exposure, an increase in the ozone exposure time to 15 min completely abrogated infectious virus copies (Figure 4D). As expected, no residual live virus could be detected 20 min after 25 ppm ozone exposure (Figure 4D).

### 3.4. Doubling the Ozone Concentration Reduced Exposure Time to Achieve Faster Virus Inactivation

**Figure 5:**
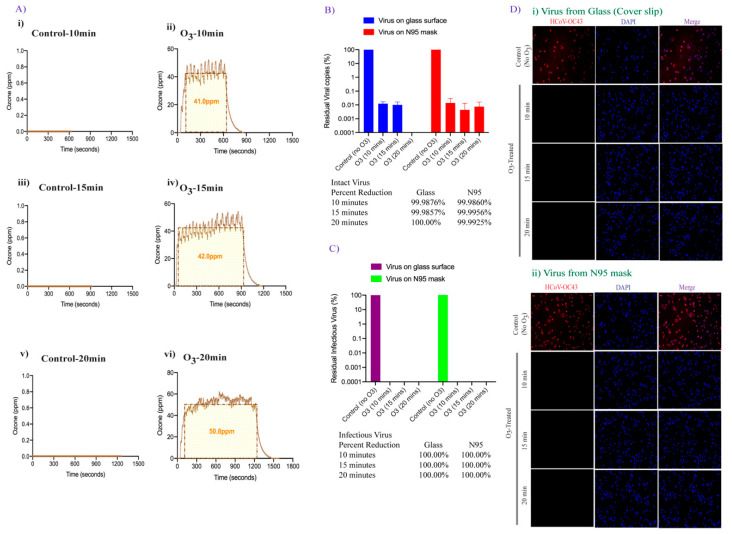
Effect of higher ozone dose on HCoV-OC43 inactivation. (**A**). The ozone (ppm) vs. time (sec) plot depicting the ozone sensor readings when the virus was subjected to ~50 ppm-10, 15, and 20 min of ozone exposure, as generated via FATHHOME device. (**B**). HCoV-OC43 virus aliquot (100 µL) was placed on glass coverslips and N95 FFRs and exposed to 50 ppm of ozone for 10, 15, and 20 min. Following ozone treatment, HCoV-OC43 was collected for viral RNA extraction, followed by quantitation of the intact residual viral copies based on the standard curve. (**C**). Following ozone exposure, HCoV-OC43 virus was collected, and subjected to the infection of A549-hACE-2 cells. Relative intracellular infectious RNA copies were calculated through qRT-PCR based on the standard curve and quantified with respect to untreated controls, set to 100%. (**D**). Uninfected and infected cells were counted using the automated Image J macro analysis. HCoV-OC43 infection was detected by immune localization of HCoV-OC43 nucleocapsid protein through IFA (red signals). Nuclei were stained with TO PRO-3 (blue signals).

We further determined whether increasing the ozone concentration can reduce the exposure time necessary for complete disinfection of HCoV-OC43 virus. To this end, the same amounts of HCoV-OC43 coronavirus were applied onto coverslips and N95 FFRs placed inside the wells of a 12-well plate and exposed to 50 ppm ozone gas and an ambient pressure of 68 kPa to 85 kPa for 10, 15, and 20 min (Figure 5A). Total RNA was extracted to quantify the intact genomic HCoV-OC43 RNA copies through qRT-PCR, as described above. As expected, prolonged exposure of the surfaces to 50 ppm ozone disintegrated 99.9% of virus genomic RNA (3-fold reduction) within 10 min of exposure, on both the tested surfaces (Figure 5B). Increasing the exposure time to 15 min did not further reduce the viral copy load detected on glass surfaces or N95 FFRs. Further increasing the exposure time to 20 min at 50 ppm ozone, resulted in no residual intact viral genomic copies detected on either glass surfaces or N95 FFRs. The recovered viruses from both the surfaces were subsequently assayed for virus infectivity using A549-ACE2 cells, and the results are shown in Figure 5C. The qRT-PCR analysis for residual live virus quantification, showed a complete disintegration of the viral genome (below the detection limit), present on both the tested surfaces, regardless of the exposure time (Figure 5C). The virus infectivity was also analyzed using IFA (Figure 5D). No residual infectious virus was detected in the virus culture assays for all the selected time points. Thus, the FATHHOME device can safely operate under OSHA and FDA ozone emissions limits at an internal ozone concentration of 50 ppm leading to 100% inactivation of HCoV-OC43 virus within 10 min.

In summary, we demonstrated that the decontamination environment provided by FATHHOME’s portable ozone-based “dry sanitizer” is highly effective in inactivating human coronavirus, HCoV-OC43, by reducing the viral genomic RNA stability and virus infectivity. We confirmed that even a brief ozone exposure, 20 ppm for 10 min, can effectively eliminate 99.8–99.9% of infectious virus on solid and porous surfaces, while maintaining ambient ozone concentrations below 0.01 ppm during the entirety of the sanitization cycle. In summary, this study provides conclusive experimental evidence that FATHHOME’s ozone-based disinfection results in a 6-log reduction in viral viability and is suitable for rapid decontamination of various environmental surfaces, PPE, and personal items, containing virus in the presence of biological fluids.

## 4. Discussion

In the past few years, four major respiratory viruses, including Influenza A viruses, SARS-CoV, MERS-CoV, and now SARS-CoV-2, have led to global infectious disease outbreaks and an international public health emergency. These epidemics/pandemics include Spanish influenza (1918), Asian influenza (1957), Hong Kong flu (1968), novel influenza A virus (2009), SARS-CoV (2003), MERS-CoV (2012), and novel SARS-CoV-2 (2019) [53,54]. Interestingly, the airborne transmission of respiratory viruses through direct or indirect contact or even without contact over a distance contributes to the associated respiratory infections being ubiquitous [55]. The widespread dissemination and survival of respiratory viruses on infected surfaces or individualized items, and the severity of infection, highlight the need of reviewing available as well as establishing newer decontamination approaches to control the spread of infections. Although masks, personal protective equipment, distancing, and disinfecting appear effective in controlling exposure to infections, the ongoing SARS-CoV-2 pandemic and potential future epidemics call for the development of new viral deactivation technologies to help control the virus spread. Thus, an urgent need remains to develop and validate virus deactivation technologies to provide rapid, effective, and safe decontamination solutions. Hence, in this study, we validated the surface inactivation of human coronavirus HCoV-OC43, a less-pathogenic model of SARS-CoV-2, using FATHHOME’s high-volume, eco-friendly, ozone-based dry sanitizing system.

Ozone, a triatomic oxygen molecule, is a potent oxidizer shown to possess anti-microbial properties [56]. Ozone is created via dissociation of oxygen molecules into atomic oxygen, which then quickly combines with other oxygen molecules to form ozone [57]. Ozone is an effective disinfectant against bacteria [58,59] and viruses [60,61] including enveloped HCoVs [62,63,64,65]. A study on efficacy of ozone solution disinfectant in inactivating SARS-CoV has shown a high dose of ozone (27.73 mg/L) could kill the virus in 4 min [66]. Anti-microbial activity of ozone towards virus inactivation of surfaces has also been demonstrated using bacteriophages ϕX174, MS2, ϕ6 and T7, as surrogates of mammalian viruses [62], and nosocomial bacterial pathogens including Clostridium difficile, Acinetobacter baumannii, and Staphylococcus aureus [67]. Although the mechanism of anti-viral activity of ozone is yet to be fully understood, ozone deactivates virus through both the direct (ozone) and/or indirect (generation of reactive oxygen species/ROS) reaction mechanisms [68]. The viral proteins of HCoVs are susceptible to oxidative damage by ozone due to the presence of oxidation-prone amino acids (tryptophan, methionine, and cysteine due to sulfhydryl residues, R-S-H), and the fatty acids (arachidonic acid, linoleic acid, and oleic acid due to unsaturated bonds) alterations, which affect the viral protein’s structure, biochemical activity and virus growth properties. Fragmentation of viral proteins allows ozone to attack and damage viral nucleic acid making it non-infectious [69]. Due to its excellent oxidizing properties, aqueous ozone has been extensively used to disinfect water, purify air, and preserve food [70], however the use of ozone gas on a commercial level still needs to be explored. Ozone in its gaseous state allows better penetration and inactivation of contaminated surfaces (both porous and nonporous) more effectively than liquid disinfectants.

Interestingly, ozone gas generated via FATHHOME’s dry sanitizing system effectively inactivated HCoV-OC43 (>99% reduction) under all tested conditions. Recently, Dubuis et al. have demonstrated that maximum anti-viral efficacy of ozone against airborne viruses requires a low dose of the ozone gas exposure combined with a high relative humidity [64]. In another study, aimed to design a mobile ozone generator for deactivating viruses, 20–25 ppm ozone coupled with >90% relative humidity was found effective in achieving at least three log_10_ virus reduction in all 12 tested viruses [71]. In both studies, the effect of the increased relative humidity was studied and shown to augment the virucidal properties of ozone. In our study, we have demonstrated that HCoV-OC43 is susceptible to oxidative damage by ozone. An interesting finding is that using FATHHOME’s device 20–25 ppm ozone exposure for 10–15 min, can completely eliminate HCoV-OC43 virus on both coverslips and N95 FFRs (more than 6 log_10_ reduction) even at ambient relative humidity (45%). Since FATHHOME can maintain ozone’s antiviral efficacy in a typical air-conditioned environment (25 °C and 45% relative humidity), it may find important and broader practical applications.

Surgical masks and N95 FFRs prevent workplace exposure to SARS-CoV-2 in essential healthcare and non-healthcare settings [43]. Concerned by the pandemic potential of COVID-19, use of face masks, surgical masks (to filter infectious particles spreading via droplets), and N95 FFRs (to filter at least 95% of particulates), became mandatory in nearly all COVID-19 affected countries. A rise in the global demand has resulted in a shortage of disposable surgical masks and N95 FFRs. Consequently, reuse of single-use disposable masks has been implemented. There is no standard decontamination protocol for safe decontamination and reprocessing of the masks, although UVGI, moist heat, microwave-generated steam, and vaporized hydrogen peroxide seem to be the current standard for N95 decontamination [33,37,39,40,41,42]. Since all these methods have advantages and disadvantages, a careful consideration of the type of respirator and biological target has to be performed, to restore the fit and filtration capacity of the N95 respirator post decontamination. In this regard, several recent reports have evaluated the effect of ozone treatment on the filtration performance of N95 respirators [46,72,73,74]. Interestingly, neither higher ozone concentration (200 ppm for 90 min) nor prolonged ozone exposure (20 ppm for 36 h) appear to degrade or damage the filtration efficiency of N95 FFRs, illustrating the effectiveness of ozone treatment for contaminated FFRs regeneration [74].

FATHHOME’s dry sanitizing technology can quickly and gently sanitize articles not compatible with traditional chemical-based disinfectants, “wet” sanitization methods, or methods involving heat and pressure. Such technology is particularly relevant to frontline workers outside the hospital setting (smaller medical/dental offices, EMS, fire and police stations, home care nurses, staff at long term care homes, retail workers, the food and hospitality industry) that produce significant amounts of daily medical and PPE waste to keep their workforce safe, but do not have access to expensive industrial-scale sanitization systems. In addition, workers are now faced with “decontamination fatigue” as time-consuming (and often ineffective) decontamination tasks, which they thought were temporary, have now become part of daily routines.

Until now, FDA has issued 14 emergency use authorizations (EUAs) allowing the emergency use of decontamination systems to regenerate N95 FFRs for reuse as a response to pandemic [75]. These disinfection systems mostly use VHP or UVGI (Lumin LM3000) to disinfect compatible N95 respirators. Major concerns during the regeneration of N95 FFRs include effective inactivation of the targeted pathogens and maintenance of the FFRs filtration performance. As ozone inactivates virus on N95 respirators with no negative impact on the fit and filtration efficiency, it offers a viable alternative to current VHP and UVGI approaches that have received FDA EUAs.

In the past 25 years, 15 major global outbreaks of airborne infectious diseases have occurred, including notable outbreaks of Influenza H1N1 in 2009 and the SARS and MERS coronaviruses in 2002–2003 2012, respectively. In a world of 7.8 billion people, environmental changes, inadequate global access to healthcare, and an interconnected global marketplace suggest that the emergence of SARS-CoV-2 will not be the last pandemic. The adoption of accessible, easily deployable, rapidly scalable, and environmentally conscious decontamination systems will balance the need for safety against the challenges of a post-pandemic world.

## Data Availability

The data supporting the findings of this study are available within this article Uppal *et al*. Pathogens.
